# Electrospun Membranes Based on Quaternized Polysulfones: Rheological Properties–Electrospinning Mechanisms Relationship

**DOI:** 10.3390/polym16111503

**Published:** 2024-05-25

**Authors:** Anca Filimon, Diana Serbezeanu, Adina Maria Dobos, Mihaela Dorina Onofrei, Alexandra Bargan, Daniela Rusu, Cristina Mihaela Rimbu

**Affiliations:** 1 “Petru Poni” Institute of Macromolecular Chemistry, Grigore Ghica Alley 41A, 700487 Iasi, Romania; diana.serbezeanu@icmpp.ro (D.S.); necula.adina@icmpp.ro (A.M.D.); mihaela.onofrei@icmpp.ro (M.D.O.); anistor@icmpp.ro (A.B.); rusu.daniela@icmpp.ro (D.R.); 2 Department of Public Health, University of Life Science Iasi, 8 Mihail Sadoveanu Alley, 707027 Iasi, Romania; crimbu@yahoo.com

**Keywords:** polysulfone-matrix composites, electrospun membranes, rheological parameters, surface properties, antimicrobial activity

## Abstract

Composite membranes based on a polymer mixture solution of quaternized polysulfone (PSFQ), cellulose acetate phthalate (CAP), and polyvinylidene fluoride (PVDF) for biomedical applications were successfully obtained through the electrospinning technique. To ensure the polysulfone membranes’ functionality in targeted applications, the selection of electrospinning conditions was essential. Moreover, understanding the geometric characteristics and morphology of fibrous membranes is crucial in designing them to meet the performance standards necessary for future biomedical applications. Thus, the viscosity of the solutions used in the electrospinning process was determined, and the morphology of the electrospun membranes was examined using scanning electron microscopy (SEM). Investigations on the surfaces of electrospun membranes based on water vapor sorption data have demonstrated that their surface properties dictate their biological ability more than their specific surfaces. Furthermore, in order to understand the different macromolecular rearrangements of membrane structures caused by physical interactions between the polymeric chains as well as by the orientation of functional groups during the electrospinning process, Fourier transform infrared (FTIR) spectroscopy was used. The applicability of composite membranes in the biomedical field was established by bacterial adhesion testing on the surface of electrospun membranes using *Escherichia coli* and *Staphylococcus aureus* microorganisms. The biological experiments conducted establish a foundation for future applications of these membranes and validate their effectiveness in specific fields.

## 1. Introduction

The biomedical domain, an interdisciplinary field that includes expertise from medicine, biology, biochemistry, and materials science, is characterized by ongoing advancements in various applications. These include the progressive refinement of biocompatible implants and medical devices, such as bone implants, contact lenses, stents, surgical sutures, and dialysis membranes. Furthermore, there have been notable strides in imaging technology, regenerative tissue engineering, and the development of drug delivery systems within this field [1,2]. For this reason, in recent decades, the biomaterials field has seen fast progression due to the demand for medical products caused by the expansion and aging of the population [3,4,5,6]. Based on a comprehensive global industry analysis report, the worldwide biomaterials market surpassed USD 94 billion in 2018. Projections indicate that it is poised to exceed USD 256 billion by 2025, reflecting a growth rate of over 15% between 2019 and 2025 [7]. 

In earlier years [8], biomaterials were frequently used as porous scaffolds, mainly prepared by solvent casting. Technological advancements have led to the adoption of new techniques, such as electrospinning, additive manufacturing, microfabrication, and bioprinting [9,10], for the preparation of porous scaffolds with intricate structures. These modern processing and manufacturing technologies enable the fabrication of bioactive scaffolds, including implants, grafts, membranes, and other biomedical devices, with specific structures to meet the urgent demand for materials in the field.

Electrospinning, a versatile and widely studied technique, holds significant promise in the fabrication of nanofibrous materials with diverse applications ranging from biomedical engineering to environmental remediation [11,12,13,14,15] due to their favorable properties, low cost, and facile production [16,17]. At the center of this process lies the intricate interplay between the rheological properties of polymer solutions and the resultant fiber morphology [18]. Understanding and optimizing these properties are paramount for tailoring the structural and functional characteristics of the electrospun fibers to meet specific application requirements [19]. Additionally, the properties and functionality of the resulting nanofibrous materials can be tailored in accordance with the purpose of their use if special attention is paid during the selection of polymer materials. Consequently, the fabrication of nanomaterials via electrospinning leads to a significant improvement in the design material used and, implicitly, in the nanomaterials achieved.

Polymers, originally designed for diverse industrial applications, have been repurposed as biomaterials due to their distinctive characteristics, such as wettability, chemical composition, porosity, flexibility/stiffness, and roughness, which are crucial in relation to the physiological environment [20,21]. Consequently, the challenge lies in innovating new polymeric materials with sustainability, multifunctionality, and advanced properties. This research aids in enhancing our comprehension of the interactions within systems and the interplay between the structure, composition, and surface properties of polymeric materials. Since most biological reactions occur on surfaces and at interfaces, a material’s performance in a biological environment depends on its surface and bulk properties. Hence, a material’s surface, determined by its distribution, functionality, and hydrophilic/hydrophobic balance [22], serves as the primary factor influencing its biological response and thus biocompatibility [23]. 

Meeting these requirements necessitates structural modifications to polymeric materials. In this direction, based on the perspectives obtained from the structure–property correlation [24,25,26], various polysulfones (PSFs) have been molecularly tailored through chloromethylation (CMPSF) and quaternization (PSFQ) reactions [27]. It is known that PSFs are the most commonly used membrane material in biomedicine for hemodialysis as well as in extracorporeal and biomedical devices [28,29,30]. Thus, functionalized polysulfones containing quaternary ammonium side groups (PSFQ), renowned for their remarkable thermal stability, chemical resistance, and mechanical strength, are widely used in various industrial and biomedical applications [23,31] as a result of their reactive groups. Although these functionalized polysulfones have excellent properties for film/membrane formation, acting as excellent barriers in biomedical applications, they have also proven to be good candidates for the development of next-generation nanofibrous scaffolds. Their inherent qualities make them suitable for fabricating nanofibrous materials with enduring durability and structural integrity. Within electrospinning, PSFQ offers engineers and researchers a versatile platform for crafting membranes, filtration systems, and biomedical implants capable of withstanding demanding environments.

On the other hand, to enhance the performance of practical electrospun material, the design of blends/composites based on PSFs/functionalized PSFs using diverse compounds is favored. Composite membranes comprise two materials with distinct characteristics: one providing physicochemical resistance and the other conferring selectivity. Hence, to alter the inherent properties of PSFs/functionalized PSFs, conventional biodegradable polymers like cellulose and its derivatives (e.g., cellulose acetate phthalate (CAP)) and polyvinylidene fluoride (PVDF) have garnered attention and interest [32,33,34]. Each brings its own unique set of characteristics to the electrospinning process, improving the properties of the material and, implicitly, the practical applications. In this context, cellulose acetate phthalate (CAP), derived from the abundant natural polymer cellulose, is notable due to its physicochemical properties (biodegradability, optical clarity, and chemical resistance [35]) and bioactivity [36]. These attributes make it a suitable material for applications demanding both environmental compatibility and mechanical performance. This versatility, depending on the targeted application, is mainly due to the presence of functional groups, which confer a high level of chemical reactivity, flexibility, and hydrophilicity. In electrospinning, CAP finds utility in the creation of nanofibers tailored for diverse applications, from tissue engineering scaffolds to filtration membranes and drug delivery systems [37]. Moreover, PVDF, distinguished by its exceptional chemical resistance, piezoelectric properties, and wide temperature tolerance, appears as a versatile material with applications in energy storage but also sensor technologies [38]. This polymer offers a unique opportunity to fabricate nanofibrous structures with specialized functionalities, such as electrical conductivity, mechanical resilience, and chemical stability [39,40]. These properties make PVDF-based nanofibers ideal candidates for applications in advanced filtration systems, energy storage, and sensing devices [41,42]. All these characteristics have an impact on the performance of the polysulfones in biomedical applications, and for this reason, there is great trust in their use in improving the properties of electrospun membranes applicable in dialysis.

In the literature, little attention has been paid to the direct blending of ionic polymers, namely PSFQ with CAP and PVDF. Therefore, it is of interest to find out if the functionalized polysulfones can be designed for specific applications in blends with PVDF and CAP and to establish their impact on different properties. For this reason, unlike previous studies on obtaining membranes based on polysulfones by applying the solution casting technique [24,25,26], the challenge of the present study consists in the possibility of designing advanced nanofibrous materials tailored to meeting the demands of diverse biomedical applications (e.g., as extracorporeal membranes) through an exploration of studied polymers (PSFQ, CAP, and PVDF) using the electrospinning technique. Consequently, the need for technological innovation to fabricate novel fibrous membranes with applications in various separation and purification processes, such as water filtration, evaporation, and dialysis, due to their highly packed density and easy management as self-supporting membranes [43], can not be ignored. Thus, the current paper studies in depth the connection between the solution’s rheology and electrospinning, with a specific focus on elucidating the mechanisms that govern nanofiber formation. By evaluating the rheological properties of polymer solutions and their impact on electrospinning outcomes, the information obtained from this research can significantly help in the design and fabrication of nanofibrous materials for various applications in the biomedical field. Hence, the current study provides insights into the future use of PSFQ/CAP/PVDF blends as electrospun membranes for hemodialysis applications.

## 2. Materials and Methods

### 2.1. Materials

**Scheme 1:**
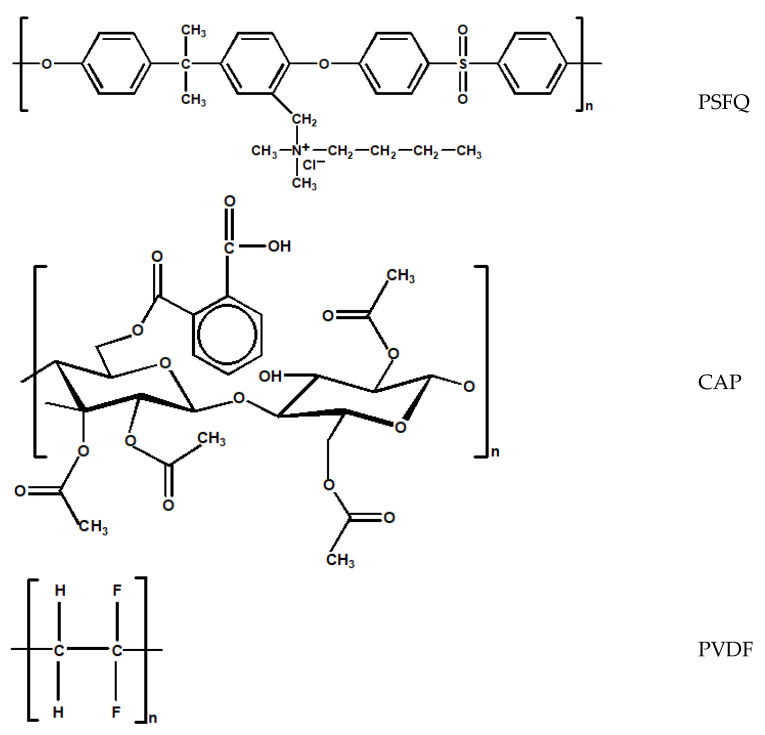
General chemical structures of functionalized polysulfone containing quaternary ammonium side groups (PSFQ), cellulose acetate phthalate (CAP), and polyvinylidene fluoride (PVDF).

A functionalized polysulfone containing quaternary ammonium side groups (PSFQ, Mn = 28,000 g/mol) was synthesized by substituting the chlorine atoms of a chloromethylated polysulfone (CMPSF, Mn = 29,000 g/mol, chlorine content, Cl = 7.42%) with ammonium groups using N,N-dimethylbutylamine tertiary amine, as described in previous studies [31,33]. Cellulose acetate phthalate (CAP, Mn = 2534 g/mol, high purity ≥99.5%, Sigma Aldrich, St. Louis, MO, USA) and polyvinylidene fluoride (PVDF, Mw = 180,000 g/mol) were obtained from Sigma-Aldrich (Sigma-Aldrich Chemie GmbH, Taufkirchen, Germany) and used as received without further purification (Scheme 1). 

N,N-dimethylformamide (DMF, Sigma Aldrich, Darmstadt, Germany), a polar aprotic solvent with high chemical polarity (dielectric constant = 37.51), a boiling point of 124 °C, a surface tension of 30.84 mN/m, and a viscosity of 1.72 cP, was employed as a good solvent for PSFQ and CAP in electrospinning, resulting in uniform fibers. *N*-methyl-2-pyrrolidone (NMP, Sigma Aldrich, Darmstadt, Germany), another dipolar aprotic solvent similar to dimethylformamide and dimethyl sulfoxide, with a viscosity of 1.65 cP, a boiling point of 203 °C, and a surface tension of 40.1 mN/m, was chosen for its ability to dissolve various polymers, including polyvinylidene fluoride (PVDF). Gram-positive Staphylococcus aureus (*S. aureus*, ATCC 25923) and Gram-negative Escherichia coli (*E. coli*, ATCC 25922) were employed as test microorganisms.

### 2.2. Preparation of the Electrospun Membranes

To obtain electrospun membranes, homogeneous solutions of PSFQ and CAP with different concentrations, varying between 15 and 40 g/dL, were prepared by dissolving them in N,N-dimethylformamide (DMF) at room temperature. At the same time, a solution of PVDF in N-methyl-2-pyrrolidone (NMP) was obtained (keeping the same concentrations) by heating it at a constant temperature of 50 °C in a water bath under continuous stirring for 4 h. Finally, the solutions were kept at rest for 24 h to achieve their degassing. In the next step, the homogeneous solutions, obtained according to the protocol mentioned above, were mixed in different ratios, thus obtaining different PSFQ/CAP/PVDF compositions (wt./wt./wt.): 70/25/5; 60/30/10; 45/40/15; 20/65/15; 40/10/50; 25/25/50; and 20/20/60.

### 2.3. Measurements

#### 2.3.1. Electrospinning Process

The electrospinning of the studied solutions was carried out using a Fluidnatek^®^ LE-50 laboratory line, delivered by Bioinicia S.L. from Valencia, Spain. The equipment was provided with a versatile high-voltage power supply that allowed for voltage adjustments within the range of 0 to 30 kV. For this particular experiment, a voltage between 21.8 and 24.2 kV was applied. The distance between the needle’s tip and the collector was set at 20 cm, but for the 25PSFQ/25CAP/50 PVDF (wt./wt./wt.) blend, it was necessary to use a distance equal to 16 cm and a flow rate for the solution in the range of 5–50 µL/min. For electrospinning, the solution was loaded into a 5 mL plastic syringe fitted with a 25G stainless steel needle, featuring an internal diameter of 0.5 mm and serving as the nozzle for the process. The resulting fibers were deposited onto a backing foil sheet affixed to a copper grid acting as the collector. Electrospinning was performed at room temperature for a duration of 2 h.

#### 2.3.2. Rheological Analysis

The rheological behavior of the PSFQ/CAP/PVDF solutions was investigated using a CS50 Bohlin rheometer (Malvern Instruments, Worcestershire, UK) equipped with a cone-plate measuring system (cone angle of 4° and diameter of 40 mm). Shear viscosity measurements were conducted over a shear rate range of 0.07–1000 s^−1^ at a temperature of 25 °C. For oscillatory shear analysis, amplitude sweep tests were initially performed at a frequency of 1 Hz across a strain range of 0.1–100 Pa. A shear stress of 2 Pa was then selected from the linear viscoelastic domain. Oscillatory shear measurements were conducted in the frequency domain of 0.1–100 Hz. Rheological tests were repeated, with measurements recorded with an accuracy of ±5%.

#### 2.3.3. Fourier Transform Infrared Spectroscopy (FTIR)

Fourier transform infrared spectroscopy (FTIR) analyses were conducted at room temperature using a Bruker Vertex 70 spectrometer (Bruker Optics, Ettlingen, Germany), equipped with a diamond crystal and a single reflection at an incidence angle of 45°. Spectra were recorded in the range of 4000–600 cm^−1^ at a resolution of 4 cm^−1^.

#### 2.3.4. Scanning Electron Microscopy (SEM)

Scanning electron microscopy (SEM) studies on electrospun fibers were performed with a Verios G4 UC Scanning Electron Microscope (Thermo Scientific, Brno, Czech Republic). To ensure electrical conductivity and to impede charge buildup during electron beam exposure, the samples were coated with a 6 nm layer of platinum by using a Leica EM ACE200 Sputter coater, Vienna, Austria. SEM examination was conducted in High Vacuum mode using a secondary electron detector (Everhart-Thornley detector, ETD) and an accelerating voltage of 5 kV. An Image J software program (Version 1.46, 14 December 2018) was used to define the diameters of the electrospun fibers. For each sample, a minimum of 25 electrospun fibers were considered to obtain the average diameter, ensuring a comprehensive and accurate analysis.

#### 2.3.5. Contact Angle Measurements

To assess the wettability of the samples, static contact angles were determined using the sessile drop method at ambient temperature, employing a CAM 101 Optical Contact Angle instrument (KSV Instruments Ltd., Helsinki, Finland). Images were captured using a specialized optical system equipped with a CCD camera and connected to a computer. Three test liquids (MilliQ water (W), methylene iodide (CH_2_I_2_), and ethylene glycol (EG)) were employed for determinations. Approximately 1 μL of liquid was deposited onto the film surface using a Hamilton syringe on a prepared substratum plate, and the resulting image was directly transmitted via the CCD camera to the computer for analysis. All experiments were conducted in triplicate, and the results were recorded as the average ± standard deviation.

#### 2.3.6. Water Sorption Capacity

The dynamic moisture sorption capacity of the PSFQ/CAP/PVDF fibrous membranes was evaluated using the fully automated gravimetric device IGAsorp, manufactured by Hiden Analytical, Warrington, UK. The key component of this device is its ultrasensitive software-controlled microbalance, which enables the measurement of weight variation as humidity is adjusted at a constant temperature. Before sorption measurements, the samples were dried at 25 °C in flowing nitrogen (250 mL/min) until the sample weight reached equilibrium at RH < 1%. Subsequently, the relative humidity (RH) was gradually increased from 0 to 90% in 10% humidity steps. Each sample was allowed to reach equilibrium for a pre-defined time between 40 and 60 min at each RH step. Finally, the RH was decreased, and desorption curves were recorded.

#### 2.3.7. Antimicrobial Activity

The quantitative determination of the antimicrobial efficacy was carried out on samples 25PSFQ/25CAP/50PVDF (A1), 40PSFQ/10CAP/50PVDF (A2), 40PSFQ/10CAP/50PVDF + α-tocoferol (A2.1), and 40PSFQ/10CAP/50PVDF + α-lipoic (A2.2). The antimicrobial activity was evaluated against *Staphylococcus aureus* (a Gram-positive species) and *Escherichia coli* (a Gram-negative species). In the practice of conventional testing, ATCC (American Type Culture Collection) reference bacterial strains are used, which are recommended in accordance with the specifications of the international standards (Clinical and Laboratory Standards Institute and European Committee on Antimicrobial Susceptibility Testing). Suspensions of the 24 h bacterial cultures were prepared with a cell density of 1.5 × 10^8^ CFU/mL saline solution, corresponding to a turbidity of 0.5 McFarland standard, which was determined using the Biosan DEN-1 densitometer. The samples were 1 cm^2^ in size and were placed in sterile vials in which 1 mL of the bacterial suspension was distributed. The contact times between the suspension and the sample were set at 6 h (T1), 24 h (T2), and 48 h (T3). The positive control (T0) was represented by the plate on which 1 mL of the 0.5 McFarland bacterial suspension (1.5 × 10^8^ CFU) was spread. The samples were incubated at 37 °C under aerobic conditions. After 6, 24, and 48 h of contact time, 1 mL of the bacterial suspension in which the samples were immersed was removed and distributed onto sterile Petri plates, over which the melted and cooled Mueller Hinton Agar (Oxoid, Basingstoke, UK) bacterial culture medium was placed at 45 °C. After solidification, all plates were incubated under the same conditions (24 h/37 °C). In the last stage, the bacterial colonies (CFU, colony-forming units) were counted and converted into logarithmic values. The antimicrobial effect was evaluated by comparing the logarithmic values obtained in the test samples with the known value of the control sample and determining the degree of logarithmic reduction. A reduction of 1 log corresponds to a reduction in bacteria of 90%, while a reduction of 5 log corresponds to a reduction of 99.999%.

## 3. Results and Discussion

### 3.1. Role of Rheological Parameters in the Electrospinning Process and Their Impact on Fibrous Membrane Characteristics

According to the literature [44], the properties of polymer solutions play an essential role in the electrospinning process, having an impact on the structural features and morphology of the resulting electrospun fibers. These system characteristics are closely related to the solution’s concentration and the solvent’s nature. Therefore, polymer solutions require sufficient chain overlap and entanglements to achieve the optimum viscosity for producing high-quality electrospun fibers [44]. In this context, the present study aims to provide a systematic explanation regarding the impact of the rheological properties of solutions on the electrospinning process. This entails, firstly, evaluating the properties of the multicomponent system (PSFQ/CAP/PVDF) solution, with a particular focus on solution rheology, and, consequently, predicting the nanofiber formation based on the rheological properties of polymer solutions. Secondly, the study aims to elucidate the morphology of the nanofibers formed through the electrospinning process. Thus, regarding the design of the polysulfone nanofibers, the effects of the polymer’s concentration and the solvent’s nature on the fiber’s formation process were considered. In addition, to select the optimum conditions for the electrospinning process, the viscosity of the polymer solutions in DMF and NMP was determined in stationary shear conditions.

**Figure 1:**
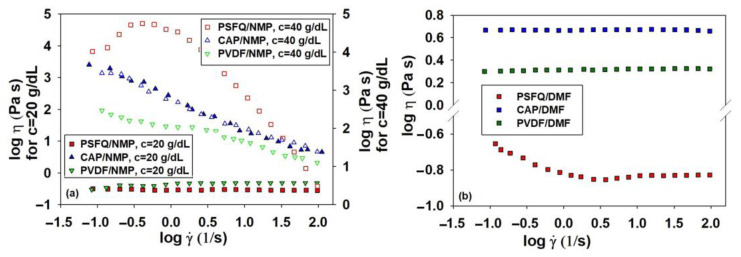
Log–log plots of dynamic viscosity (η) as a function of shear rate ($\dot{\gamma }$) at 25 °C for (**a**) pure polymer solutions (PSFQ, CAP, PVDF) in NMP at different concentrations and (**b**) pure polymers in DMF at a concentration of 40 g/dL.

**Figure 2:**
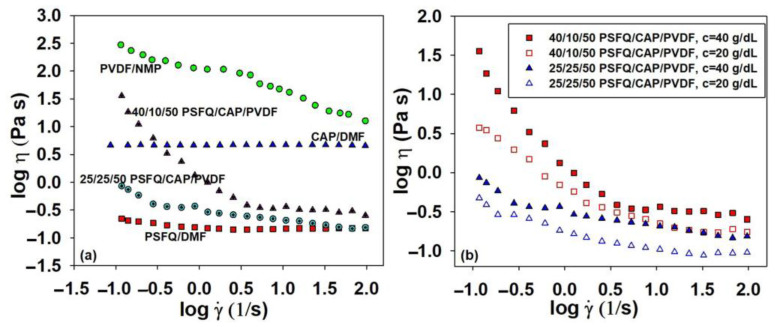
Log–log plots of dynamic viscosity (η) as a function of shear rate ($\dot{\gamma }$) at 25 °C for (**a**) pure polymer solutions of PSFQ in DMF, CAP in DMF, and PVDF in NMP and their blends in a 50/50 (*v*/*v*) DMF/NMP solvent mixture for a 40 g/dL concentration and (**b**) 25/25/50 and 40/10/50 PSFQ/CAP/PVDF (wt./wt./wt.) blends at concentrations of 20 and 40 g/dL, respectively.

Since the viscosity of the polymer solution is one of the important factors in electrospinning, in Figure 1 and Figure 2, the results obtained from the rheological tests are presented. Indeed, changes in viscosity as a function of the shear rate for the solutions of the pure polymers and their blends in different mixing ratios occur due to their complex behavior under the specific characteristics of each polymer within the blend. The analysis of the rheological curves showed differences in rheological behavior between the polymeric solutions; thus, in the case of PSFQ in NMP, the shape of the viscosity curves is identical for concentrations between 15 and 35 g/dL, the behavior being Newtonian when viscosities are independent of the applied shear rate, and the viscosities increase as the concentration of the solution increases (Figure S1). An exception occurs for higher concentrations of 40 g/dL, where the viscosity values decrease with an increase in shear rate, indicating that all the solutions behave as non-Newtonian pseudoplastics, being shear-thinning fluids (Figure 1a).

Instead, by examining the flow curves obtained in DMF, such as, for example, for the PSFQ solution with a 40 g/dL concentration, the shape of the rheological curve clearly shows a gradual decrease in the viscosity value with increasing shear rate, indicating that the solution behaves as a shear-thinning fluid, followed by a region with constant values, a Newtonian plateau, in the logarithmic range of shear rates between 0.4 and 2.0 s^−1^ (Figure 1b). These findings suggest that the pronounced shear-thinning effect may be attributed to the structure of the polymer chains in the solutions, with the solutions’ pseudoplasticity increasing as a result of the polyelectrolyte effect. As indicated in the literature [45,46], non-Newtonian behavior can be explained by the tendency of applied force to disturb polymer chains from their equilibrium conformation, leading to elongation in the direction of shearing. In essence, the thinning behavior arises from structural changes in the material that create interactions between the polymer chains and the dispersion medium. Consequently, as the shear force progressively increases and becomes predominant, the polymer chains orient themselves in the flow direction, reducing the probability of interchain associations in favor of intrachain associations, thereby lowering the viscosity [47].

In addition to the solution’s concentration, the choice of solvent plays a crucial role in determining the rheological parameters because it affects the spinning capacity [48]. Throughout the electrospinning process, both internal and external charge forces induce whipping of the liquid jet towards the metallic collector. This whipping motion facilitates the stretching and sliding of polymer chains within the solution while the solvent gradually evaporates. Consequently, polymer fibers are randomly deposited or oriented on the collector, ultimately forming a fibrous mat consisting of fibers with submicronic diameters [49]. This aspect is more obvious in the case of the neutral polymers, CAP and PVDF, for which their rheological behavior reveals that, regardless of their characteristics, hydrophilic CAP and hydrophobic PVDF in DMF show Newtonian behavior over the entire range of shear rates, their viscosity being constant. Instead, in the case of the CAP and PVDF solutions made in NMP, at c = 40 g/dL, the systems show pronounced shear-thinning behavior without appearing in the Newtonian domain (see, for example, Figure 1a).

As mentioned, a detailed investigation of the rheological parameters of the blended solutions is necessary because complex processes appear, and these parameters differ due to the reorganization of the polymer molecules at the interface itself. The data obtained for both blended solutions show that the viscosity curves of the PSFQ/CAP/PVDF blends are located between those corresponding to the pure components, indicating the components’ miscibility and compatible one-phase blend formations (Figure 2a). The differences between the rheological behavior of the blended solutions and the pure polymers, implicitly the pronounced shear-thinning, are highlighted in Figure 2a since the shapes of the curves are dependent on the solutions’ compositions. Thus, the solution with a high content of PSFQ shows a greater decrease in viscosity with increasing shear rate relative to the pure PSFQ solution (Figure 1b and Figure 2a). These results support the conclusion that the pseudoplasticity of the solutions increases with an increasing polyelectrolyte proportion in the blends (Figure 2b).

**Figure 3:**
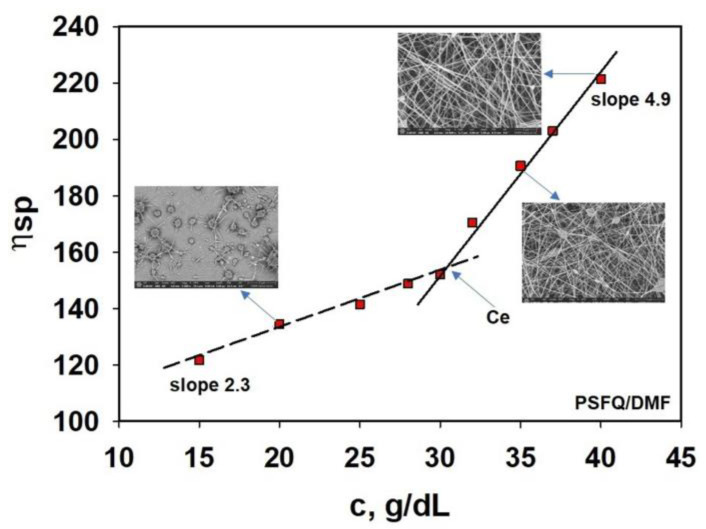
Dependence of the specific viscosity on the concentration for the PSFQ solution in DMF. SEM images were recorded for good visualization of the nanofiber’s formation.

The concentration domains for the polymeric solutions analyzed were also determined by rheological measurements using the viscosity values obtained in the Newtonian domain for different concentrations. Thus, the specific viscosity, η_sp_, was calculated as a function of the zero-shear rate viscosity, η_0_, and the solvent’s viscosity, respectively [50]. An analysis of the specific viscosity’s dependence on concentration serves as a method of determining the entanglement concentration, Ce, defined as the point of transition from the semidilute unentangled regime to the semidilute entangled regime, identified by the change in slope at the onset of the entangled regime (Figure 3, exemplified for PSFQ) [51,52]. In the case of the PSFQ sample, from the crossing point corresponding to the fitted curves from the semidilute unentangled and semidilute entangled regimes, Ce was determined to be approximatively 30–32 g/dL. The experimental data plotted in Figure 3 describe two linear dependences with slopes of 2.3 and 4.9 in the semidilute unentangled and semidilute entangled regimes, respectively. The results are in good agreement with the theoretical predictions, according to which the viscosity should increase with the polymer concentration so that the slope is approximately 4.8 [50,53].

The obtained results suggest that, for concentrations higher than 30–32 g/dL, the macromolecular chains are entangled, ensuring the optimal conditions for the electrospinning process [54,55,56], which can be confirmed by SEM images (see the small images in Figure 3). Thus, it can be observed that for concentrations lower than 30 g/dL, the increase in viscosity with increasing concentrations is slow, while for concentrations higher than 30 g/dL, a slight change occurs with the viscosity increasing significantly; in these conditions, after Ce, fiber formation occurs. This systematic increase in Ce can be explained by considering the structural properties of PSFQ, namely its charge density and the size of polycation, which hinder chain overlap; only the outermost chains can entangle with each other [57]. Therefore, the experimental data showed that beaded nanofibers were produced when the solution concentration was slightly higher or equal to Ce, and uniform fibers without beads were formed at a 40 g/dL concentration because the chain entanglement became sufficient to form nanofibers without defects.

On the other hand, in the case of the CAP sample, a weaker dependence was obtained in the semidilute entangled regime (η_sp_~1.4), while in the semidilute entangled regime, the concentration exponent was 3.2. Changes in the slope marked the onset of the semidiluted unentangled and semidiluted entangled regimes at a Ce of approximately 25–28 g/dL (Figure S2). Studying rheology in relation to electrospinning conditions emphasized the influence of carboxylic groups on the solution’s features and the fiber’s dimensions. Additionally, cellulose acetate phthalate (CAP) facilitates the apparition of hydrogen bonding, involving both carboxyl and hydroxyl groups. The intensification of these interactions depends on the solvent employed and can play a significant part in the electrospinning process. Instead, the initiation of electrospinning for the PVDF sample takes place at concentrations of about 35 g/dL due to the fact that PVDF chain entanglement numbers increase as the concentration increases. Finally, a continuous and bead-free fiber will be obtained due to the formation of an elastically deformable network. The increasing entanglement concentration at approximately 32–35 g/dL is caused by lower-molecular-weight chains occupying a smaller hydrodynamic volume, and, for this reason, a higher concentration is required before they begin to topologically constrain one another and entangle. At concentrations below the critical concentration (Ce), beaded fibers are formed. In this region, there is not enough interpenetration of the chains. These entanglements fail to stabilize the jet of the PVDF solutions when subjected to a strong elongational flow field (Figure S2).

It was established that solutions with low viscosity in the electrospinning process predominantly yield spindle-shaped seeds instead of fibers. Electrospinning from these solutions tends to be discontinuous and unsuccessful. As viscosity increases, the formation of nanofibers becomes possible, and the occurrence of spindle-shaped seeds diminishes. However, as viscosity continues to rise, spindle-shaped seeds may reappear, and fibers with various thicknesses may form, likely due to the splitting of the flow ejected from the needle to the collector. If viscosity continues to increase beyond a certain point, fiber formation does not occur because solutions with excessively high viscosity do not have the ability to get out of the syringe [58]. Based on these remarks, we can conclude that in the case of the studied polymers, at a concentration of about 40 g/dL, according to research on solution rheology in relation to the electrospinning conditions, defect-free fibers are obtained, as can be seen in the SEM micrographs. Moreover, it is necessary for solvents to have better volatility and vapor pressure and to maintain the consistency of the polymer solution. The stability of the solution jet during the process is quite good at this polymer concentration, i.e., 40 g/dL solutions of PSFQ in DMF, CAP in DMF, and PVDF in NMP. Therefore, this concentration is recommended for the electrospinning process in order to minimize the energy consumption, and, in these conditions, smooth fibers without beads are obtained. Thus, the most suitable ratio for obtaining fibers with improved structural and morphological features is a 50/50 (*v*/*v*) DMF/NMP mixture composition.

**Figure 4:**
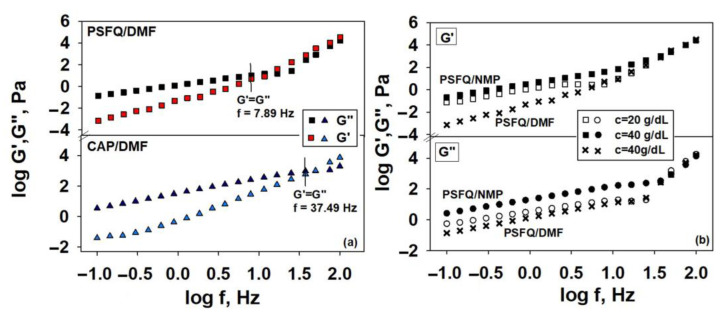
Log–log plots of shear moduli G’ and G” versus oscillatory frequency (f) at 25 °C for pure samples: (**a**) PSFQ and CAP in DMF at a 40 g/dL concentration and (**b**) PSFQ in NMP at concentrations of 20 and 40 g/dL, respectively (□,○, ■, ●), as well as PSFQ in DMF at a concentration of 40 g/dL (**x**).

The viscoelastic behavior of the concentrated solutions studied was also investigated, providing information on the degree of viscoelasticity of the polymer samples subjected to the action of a constant shear stress. In particular, the influence of the polymer’s structural characteristics and the blend composition on the macromolecular chain’s flexibility in the shear field is reflected in the storage, G′, and loss, G″, moduli. As an example, Figure 4a shows the rheological behavior for the PSFQ and CAP samples, where, in a low oscillation frequency range, the loss modulus is always higher than the elastic one, G″ > G′, which is typical behavior for viscous liquids as a result of unrecoverable viscosity loss. Instead, in the high frequency range, the storage modulus, G′, becomes higher than the loss modulus, G″. This observation is an indicator that the solid-like character becomes predominant and determines the reversibility of the energy stored in the sample as a result of the number and strength of interactions in the system [59].

Moreover, the crossover frequency values, for which G′ = G″, reflect the conformational changes/structuring of the polymer blend components, as well as the cumulative effects of electrostatic interactions, hydrogen bonding, or aggregation phenomena. Structuring is determined mainly by macromolecular interpenetration and/or by the physical interactions between the chains. Also, one can remark that the overlap frequency exhibits lower values for PSFQ in DMF but becomes higher for CAP in DMF, an aspect which indicates that the CAP content in the polymer blends enhances the system’s elasticity, reflecting the influence of flexibility in jet initiation/formation and stabilization. Another way of emphasizing the influence of the shear elasticity of the system in jet stabilization is represented by the variation in the dynamic moduli with concentration (G′ and G″ increase with increasing concentration; see Figure 4b). Our results are in accordance with the explanations from the literature, which highlight the role of the elastic response over the plastic response in jet stabilization [60,61]. In addition, our findings maximize the importance of the response of the PSFQ, CAP, and PVDF polymer chains in the composite system for the stability of the solution jet during the process; so, it should be taken into consideration that the amount of each polymer in the mixture is appropriate. Thus, according to the obtained rheological data, it is established that the minimum required proportion of polyelectrolyte from the processing solution is 40 wt.%, which is dependent on the morphology of the fibers. For stabilization, some segments of the PVDF polymer chains make inflexible contacts. This network significantly influences fiber development and prevents their breaking in the electrospinning process. For this reason, a higher proportion of PVDF (50 wt.%) was used in the processing solution.

**Figure 5:**
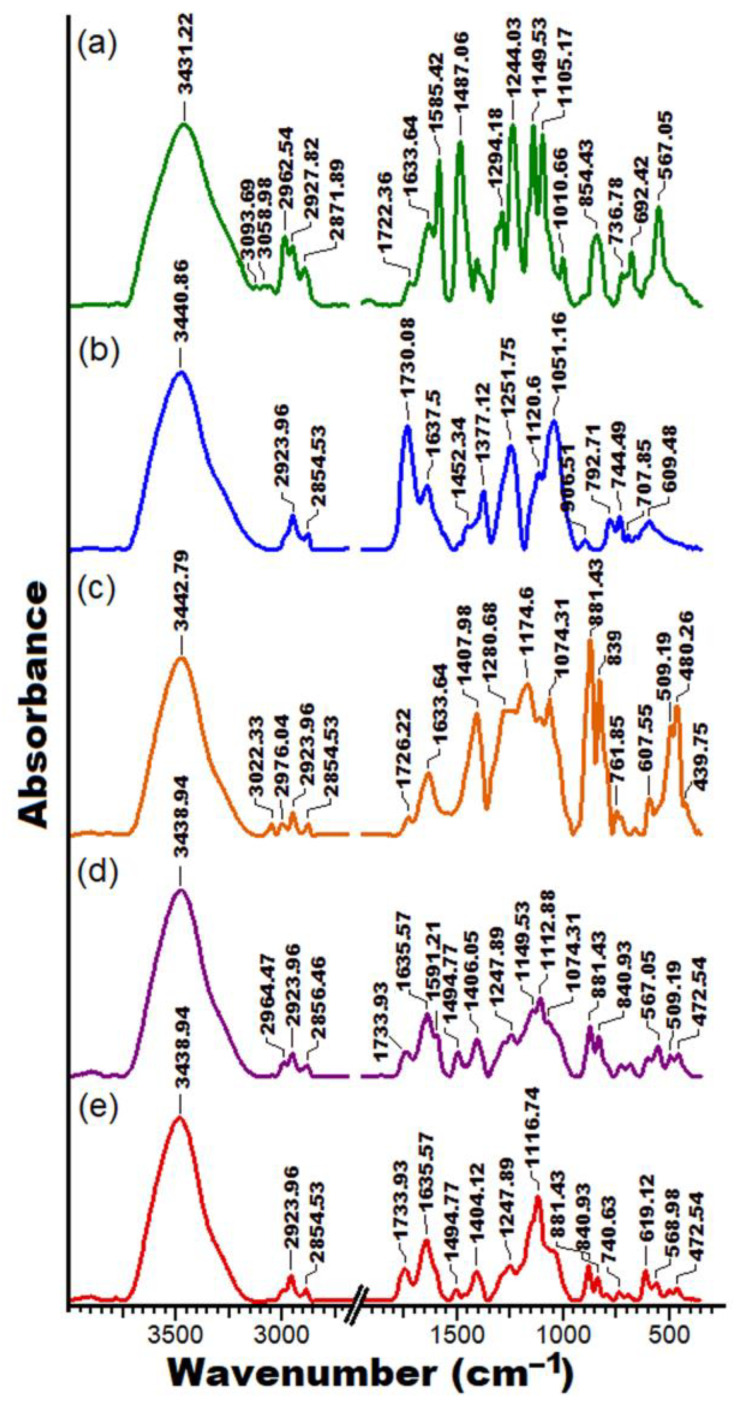
FTIR spectra for the PSFQ (**a**), CAP (**b**), PVDF (**c**), 25PSFQ/25CAP/50PVDF (**d**), and 40PSFQ/10CAP/50PVDF (**e**) samples.

As has already been shown, the properties of the polymer solutions generate significant differences from the point of view of the changes induced in the fibers obtained as a result of the functional groups as well as the chemical bonds generated by the intra- and intermolecular interactions that occur in the studied systems (Figure 5). Hence, the FTIR spectra proved effective for examining the modifications resulting from the physical interactions between the chains, as evidenced by measuring the relative intensities of the vibrational bands in the blended samples compared to those in the pure compounds. The polymer mixture samples displayed characteristics akin to those of the pure components (PSFQ, CAP, PVDF), with slight additional changes in the shape and intensity of the peaks. These alterations reflect the capacity of the compounds used to produce novel types of interactions [62,63,64,65].

Based on the results from previous studies [23,24,25,26], the FTIR analyses indicate the characteristic bands for the polymers studied. According to Figure 5a, the FTIR spectrum for the PSFQ structure displays various absorption bands assigned to the –CH_3_ and –CH_2_ vibrations of the aliphatic units at around 2962.54–2927.82 cm^−1^ and 2871.89 cm^−1^, respectively. Also, absorption bands corresponding to –SO_2_ appear around 1294.18 cm^−1^ and are attributed to asymmetric stretching, and at 1149 cm^−1^, a strong absorption band occurs corresponding to symmetric stretching. In addition, the absorption bands at approximately 1585.42–1487.06 cm^−1^ describe the presence of C=C aromatic compound vibrations on the polysulfone. A characteristic band of the quaternary ammonium group was confirmed by the appearance of stretching vibration peak at about 1633.64 cm^−1^. The results of the FTIR analysis of the CAP spectrum (Figure 5b) present particular absorption bands around 2923.96 cm^−1^ and 2854.53 cm^−1^, attributed to the symmetric and asymmetric stretching vibrations of methyl –C–H groups. Furthermore, the adsorption peaks corresponding to –C=O carbonyl group vibrations showed peaks at about 1730.08 and 1637.5 cm^−1^, while the –C=C– linkage of the aromatic ring was seen at about 1558.42 cm^−1^. In addition, the characteristic bands at 1452.42 cm^−1^ and 1377.12 cm^−1^ correspond to the stretching vibrations of –C–H bonds from methylene groups. Also, the absorption bands corresponding to the –C–O– stretching vibrations of a cyclic ether structure appeared at approximately 1120.60 cm^−1^ to 1051.16 cm^−1^.

On the other hand, from the FTIR spectrum of PVDF (Figure 5c), one can detect a typical peak around 1174.6 cm^−1^ identifying the stretching vibrations of CF_2_. Also, the characteristic absorption peak at 1407.98 cm^−1^ reveals stretching and deformation of the CH_2_ alkane bond, and the weak peaks at about 3022.33 cm^−1^ and 2976–2854.53 cm^−1^ indicate C–H stretching in the PVDF compound [62,63]. In addition, the bands observed in the PVDF spectra at 839 cm^−1^ and 1407.98 cm^−1^ are common in the β phases of PVDF [65,66], while those corresponding to 480 cm^−1^ and 881.43 cm^−1^ are associated with the amorphous phase of the polymer [67].

In the spectrum of the composite fibrous membranes, where the three polymers are found in different mixing ratios, the characteristic absorption peaks of the pure PVDF fibrous membranes were retained (Figure 5c,d). This is understandable since the ratio of PVDF in the blends is the highest. However, as can be seen, for the exemplified mixtures, considerable changes take place in terms of the shape and intensity of the peaks. In this sense, it is observed that the peaks that appear in the wavenumber range of 450–1800 cm^−1^ considerably change their shapes and intensity, with an essential decrease in their intensity being evident. Thus, the peaks at 480 cm^−1^ and 881.43 cm^−1^ attributed to the amorphous phase of PVDF as well as the peak characteristic of its β phases remain in the case of the studied mixtures but decrease in intensity with an increase in the PSFQ ratio in the systems. Also, for the 25PSFQ/25CAP/50PVDF blend, a new peak appears at a wavenumber of 1494.77 cm^−1^. This is maintained, but slightly decreases in intensity for the 40PSFQ/10CAP/50PVDF blend. The peak observed in the FTIR spectra of PVDF at 2976.04 cm^−1^ decreases in intensity and moves to a lower wavenumber of 2964 cm^−1^ for the 25PSFQ/25CAP/50PVDF blend, becoming imperceptible for the 40PSFQ/10CAP/50PVDF mixture. All these changes in the blends’ spectra appear, on the one hand, as a result of the hydrogen bonds generated by the –OH, C=O, and C–O–C groups and free/associated groups in the systems [26,68] and, on the other hand, because of the electrostatic repulsive interactions created by the polysulfone chain groups, which must also be taken into consideration, especially for blends with a high content of PSFQ.

Murray [69] observed that interpolymer interactions lead to the formation of a compact network, resulting in a hydrodynamic volume lower than the volume sum of the individual macromolecules. This phenomenon results in a decrease in the solution’s viscosity at higher shear rates (see Figure 2a). Furthermore, the influence of the viscosity of a solution with a high content of PSFQ can be related to the fact that the ionic polymer leads to a less ordered structure due to stronger repulsive forces between charged groups and favorable polymer–solvent interactions. Consequently, this analysis is useful to identify the structures generated by the presence of functional groups on the surface or inside nanofibrous membranes. An evaluation of the differences/variations observed in the shape, broadening, and intensity of the characteristic peaks of the functional groups can demonstrate how they are distributed during the electrospinning process depending on the solvents used, as well as provide information regarding subsequent stabilization in the membrane structure.

**Figure 6:**
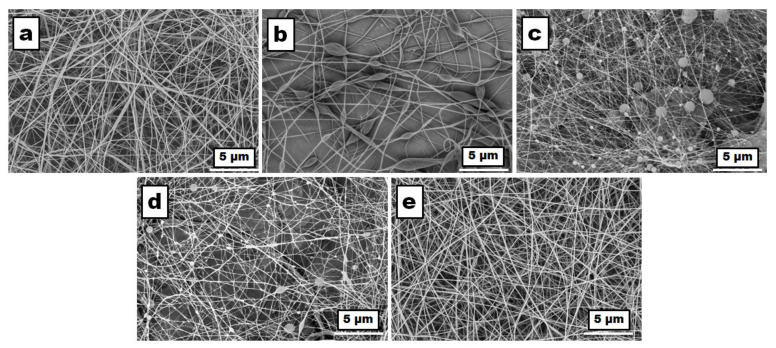
SEM images of the fibrous membranes obtained from the solutions of the pure polymers and their blends with different compositions at a concentration of 40 g/dL: (**a**) PSFQ in DMF, (**b**) CAP in DMF, (**c**) PVDF in NMP, (**d**) 25PSFQ/25CAP/50PVDF in 50DMF/50NMP, and (**e**) 40PSFQ/10CAP/50PVDF in 50DMF/50NMP, recorded with a magnification of 5000× in a 5 μm scan area.

As mentioned, identifying the key parameters essential for predicting the success of electrospinning based on the solution’s properties is crucial. In this context, the morphology, investigated by scanning electron microscopy (Figure 6), dictated by the local surface properties and geometric characteristics of the fibers (i.e., diameters and the size distribution, length, and orientation/uniformity of the fibers), density, and surface-to-volume ratio, are determining parameters in obtaining high-performance membranes that can be used in biomedical applications. To address these problems, it is essential to understand and analyze in detail the effects of all these parameters governed by the properties of the solutions used in electrospinning. Thus, when fluids have a low relaxation time or low viscosity, they tend to form beads, or defects (“beads”) [70]; this phenomenon is determined by the Rayleigh instability caused by surface tension [71,72,73,74].

However, the viscoelastic behavior of the fluid can counteract this instability, leading to the fabrication of smooth fibers instead [73]. Yu et al. [61] studied this effect and found that when the viscoelastic force completely removes or resists instability, fibers without defects are produced. This instability arises because the surface tension compels a liquid to minimize its surface area per unit mass, typically leading to a spherical shape [75].

Our investigation revealed a notable correlation between the concentration of the polymer solution or the molecular mass of the polymers and the resultant viscoelasticity. As these parameters increase, so does the viscoelasticity of the solution. This increase in viscoelasticity plays a significant role in impeding the formation of granules during the electrospinning process. Granule formation is often undesirable, as it can lead to irregularities in fiber morphology and compromise the quality of the nanofibers produced. However, it is essential to note the existence of a trade-off inherent to this phenomenon. While higher viscoelasticity effectively suppresses granule formation, it also contributes to an increase in fiber diameter. This trade-off underscores the delicate balance required to optimize the electrospinning process for the desired outcomes. Achieving a fine balance between minimizing granule formation and controlling fiber diameter is crucial for producing nanofibers with the desired properties and dimensions, thus highlighting the complexity of polymer solution rheology in electrospinning applications. Based on the data presented in Table 1 and the SEM images (Figure 6), it was observed that when the concentration of CAP in DMF was 40 g/dL, the nanostructures exhibited distinct bead formations, including spindle-shaped and concave-shaped beads (Figure 6b). In contrast, both the solutions of PSFQ in DMF and PVDF in NMP, at a concentration of 40 g/dL, resulted in fibers primarily containing concave beads. However, it is worth noting that PSFQ displayed fewer beads compared to PVDF (Figure 6a,c). By varying the combination of polymers (wt./wt./wt.), such as in the 25/25/50 and 40/10/50 compositions of PSFQ/CAP/PVDF, the surface tension increased, leading to fewer beads (Figure 6d). The increase in viscosity contributed to the absence of these bead-like structures in the membrane, a phenomenon supported by prior findings in the literature. These observations highlight the influence of polymer concentration and composition on the morphology of the resulting nanostructures. Additionally, they underscore the importance of considering various parameters, such as surface tension and viscosity, in optimizing electrospinning processes for the desired outcomes [58].

These precise measurements of average fiber diameters provide valuable insights into the influence of polymer composition on fiber morphology and size, aiding in the optimization of electrospinning processes for the desired nanofiber characteristics. Additionally, in the case where PSFQ comprises a higher proportion of the composition, more uniform fibers are evident (Figure 6e). These fibers display smooth, nonporous surfaces, and their arrangement relative to each other appears random. Consistent with expectations based on the data in Table 1, there is an approximate 66% increase in the average fiber diameter for the electrospun membrane derived from the 40PSFQ/10CAP/50PVDF solution in 50DMF/50NMP compared to the average fiber diameter obtained from the PVDF solution in NMP.

### 3.2. Control of Fibrous Membranes’ Surface Properties: Hydrophobic/Hydrophilic Balance and Water Sorption Characteristics

**Figure 7:**
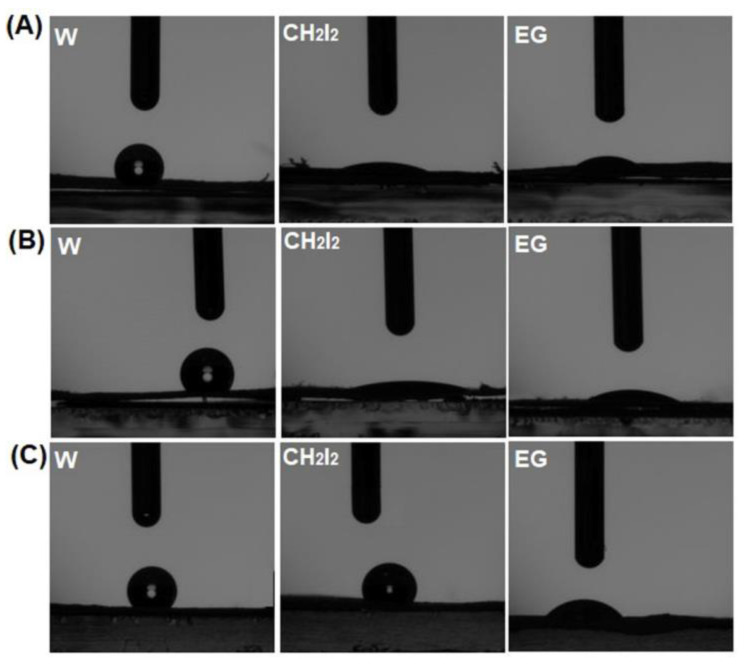
Geometric configuration of the test liquids’ drops at the interface of the PSFQ (**A**), CAP (**B**), and PVDF (**C**) fibrous membranes.

The control and establishment of hydrophobic/hydrophilic balance is of major importance, as it is the first indicator regarding the successful creation of electrospun high-performance membrane materials based on polysulfones that are applicable to solving some medical problems. In this context, the evaluation of the wettability properties of the fibrous membranes initially involved measuring the contact angle using water (W), diiodomethane (CH_2_I_2_), and ethylene glycol (EG) as test liquids (refer to Table 2). The wetting characteristics of polymer surfaces are governed by interactions occurring at the solid–liquid interface, arising from physical contact between the two phases. When a drop of liquid makes contact with a solid surface, it adopts a geometric configuration that reflects the total energy of the solid–liquid system [76,77]. The surface tension of each liquid and the interactions that are established between the fibrous surface and the liquid drop will lead to modifications in its form when it is put in contact with the polymeric surfaces (Figure 7).

Figure 7 shows that for the PSFQ, CAP, and PVDF samples, the test liquid drops change their shapes from round to flat. This means that water drops are less adsorbed by the fibrous surface than the drops of the other two liquids.

On the other hand, from Table 2, it is obvious that the contact angle values vary with the nature of the test liquids, the mixing ratio of the three components, and the fibrous membrane characteristics obtained (fiber diameter, roughness). According to the literature, samples with contact angles varying in the range of 10° < θ < 90° are considered hydrophilic, while those with contact angles within the range of 90° < θ < 150° are considered hydrophobic. Superhydrophilicity and superhydrophobicity appear when the contact angles are within the ranges of θ < 10° and θ > 150°, respectively [78,79]. Thus, taking into account these ranges, it is obvious that the samples are hydrophobic. However, by carrying out this analysis according to the nature of the polymers, it seems that the PSFQ membrane is slightly more hydrophilic compared to those obtained from CAP and PVDF, and this can be attributed, on the one hand, to the quaternary ammonium groups and, on the other hand, to the solvent’s nature.

**Table 2:** Contact angle between the different test liquids for fibrous membranes obtained from PSFQ, CAP, PVDF, and their blends (PSFQ/CAP/PVDF) in different mixing ratios.

Sample		Contact Angle (°)	
	W	CH_2_I_2_	EG
PSFQ/DMF	116.25	17.30	25.41
	79 *		
CAP/DMF	128.30	14.91	19.21
	52 *		
PVDF/NMP	132.19	102.22	43.63
25PSFQ/25CAP/50PVDF in 50/50 DMF/NMP	131.99	31.90	24.35
40PSFQ/10CAP/50PVDF in 50/50 DMF/NMP	121.95	118.56	28.23

* According to previous results obtained for PSFQ and CAP films [24,80].

As is known, the amino groups in the polymers are responsible for the polarity or hydrophilicity of the samples [81]. Also, DMF has a slightly higher polarity than NMP, and as a result, it will cause the macromolecular chains to be more flexible and result in better adsorption of water drops. At the same time, the influence of the solvent and the chemical structure of the polymers, as well as their mixing ratio, can be seen in the slight variation in the water contact angle values of the fibrous membranes obtained from the polymer blends (25PSFQ/25CAP/50PVDF and 40PSFQ/10CAP/50PVDF). Regarding the values of the contact angles measured using CH_2_I_2_ and EG, a significant decrease is generally observed as a result of the specific parameters of the liquid, especially its surface tension and forces, which they determine [76,77].

Some of the previous studies in the field [82,83] have shown that small diameters of fibers will determine high values for their contact angles, while others [83] have demonstrated the opposite effect, namely an increase in the contact angle with fiber diameters. As can be seen from the SEM images (Figure 6), in our case, for the fibrous membranes obtained from the polymer solutions in DMF (PSFQ and CAP), the contact angle values are slightly lower compared to those corresponding to the membrane obtained from the PVDF solution in NMP, which means that they are slightly hydrophilic, and the diameter of the fibers differs insignificantly. For the membranes obtained from the polymer blends, as expected, the diameter of the fibers is larger than in the case of PVDF (Table 1 and Figure 6), which is consistent with data in the literature, according to which the use of NMP as a solvent generally leads to larger nano/microfiber diameters [84]. Furthermore, the presence of non-uniformities/formations in the structure of an electrospun membrane leads to high contact angles and, implicitly, hydrophobicity. On the other hand, for the membranes obtained from the polymer blends, especially for the 40PSFQ/10CAP/50PVDF blend, these defects become less visible, and, consequently, a slight increase in hydrophilicity is observed. Despite the hydrophilicity/hydrophobicity analysis, the fiber diameter is usually not a convincing parameter unless it is correlated with the surface roughness or porosity of the membranes’ surfaces. This is supported by our previous studies or studies carried out on different compounds [24,80,85] where, unlike measurements performed on polymer films obtained through casting solutions, the contact angle values for the electrospun membranes are higher (Table 2). This means that the processing technique has a major influence on the wettability through the morphological and topological properties (roughness and porosity) that are imprinted on the surfaces of the obtained membranes.

The surface free energy, ΔG_w_, is also an important parameter by which the hydrophilic/hydrophobic character of the studied samples can be established. The variation in the fibrous membrane’s morphology (fiber diameter, the presence or absence of beads), depending on the chemical structure of PSFQ, CAP, and PVDF, as well as the ratio in which they are found in the studied mixtures, influences this parameter. Its evaluation was carried out using Equation (1), with the total surface tension of water, γ_lv_ = 72.80 mN/m [86], and θ_water_ from Table 2.

(1) $$\Delta G_{w}=-\gamma_{lv}\cdot (1+\cos\theta_{\mathrm{water}})$$

**Figure 8:**
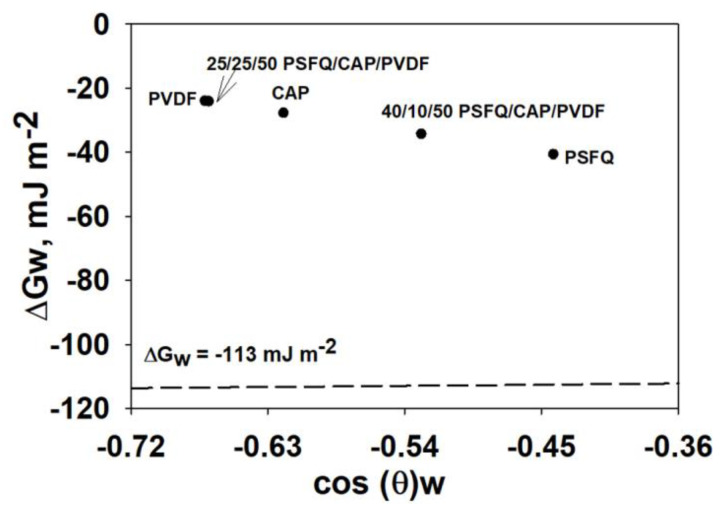
Surface free energy assessed from contact angle analysis for the studied fibrous membranes.

The literature specifies that a value of ΔG_w_ < −113 mJ/m^2^ corresponds to a hydrophilic surface, while values of ΔG_w_ > −113 mJ/m^2^ are specific to hydrophobic ones [76]. As can be observed from Figure 8, the obtained data denote a hydrophobic nature for all the fibrous membranes studied. The variations observed in the values of the surface free energy are generated, on the one hand, by the chemical structure of the polymers, their mixing ratios, and the nature of the solvent and, on the other hand, by the positioning of the functional groups on the surface of the fibrous membranes after the electrospinning process.

**Figure 9:**
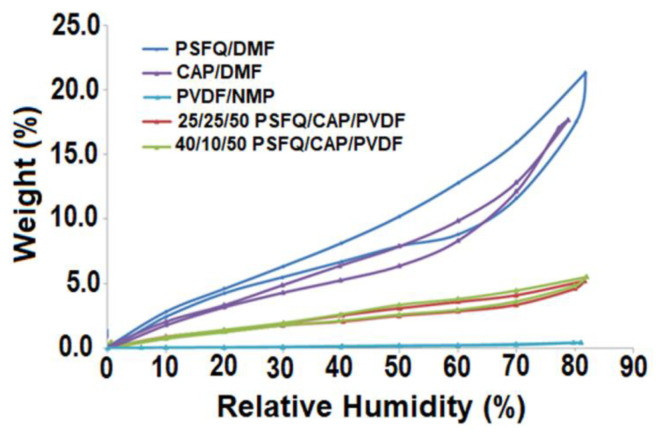
Sorption/desorption isotherms for the obtained fibrous membranes.

In addition to wettability, the sorption properties play a crucial role in determining the stability, processing, and performance of polysulfone membranes in practical applications. Therefore, to assess their suitability for practical use, the water vapor sorption capacity of these membranes was evaluated. As depicted in Figure 9 and detailed in Table 3, the permeability of polysulfone fibrous membranes is significantly influenced by their morphological characteristics, including fiber diameter and the presence or absence of “beads”, as well as their hydrophilic or hydrophobic nature.

Considering the findings from the contact angle measurements, it was anticipated that the samples exhibiting slightly higher hydrophilicity would demonstrate superior permeability characteristics. This rationalizes why PSFQ exhibits the highest permeability, while PVDF displays the lowest (Figure 9). These insights shed light on the intricate interplay between membrane morphology, surface properties, and permeability, guiding the optimization of polysulfone electrospun membranes for various applications.

Observing the shape of the water sorption curves/isotherms depicted in Figure 9, they exhibit characteristics similar to Type V isotherms, as per the IUPAC classification. This type of isotherm, accompanied by hysteresis, is commonly associated with porous surfaces, indicative of a hydrophobic material [23,24,47]. From the shape and location of the hysteresis, information regarding the surface of the samples can be obtained. The materials under investigation demonstrate minimal water vapor sorption at low relative humidity (RH) levels (0 to 10%), followed by moderate sorption at intermediate RH values. However, there is a significant increase in water absorption observed at RH values approaching 100%. In agreement with these observations, the equilibrium values of the adsorbed mass (W) at the end of each moisture stage were used to calculate the sorption and desorption isotherms, and the values of the surface parameters analyzed from the sorption/desorption isotherms for all studied systems are listed in Table 3.

The Brunauer–Emmet–Teller method (BET, Equation (2)) was used to evaluate the specific surface area based on the water vapor sorption data recorded under dynamic conditions, as presented in Table 3. The BET model describes the sorption isotherms up to a relative humidity of 40%.

(2) $$W=\frac{W_{m}CRH}{\left(1-RH)(1-RH+CRH\right)}$$

where W is the amount of water absorbed, W_m_ represents the amount of water that forms the monolayer, C is the sorption constant, and RH is relative humidity.

The average size of the pores listed in Table 3 was estimated by applying the model of Barett, Joyner, and Halenda (BJF, Equation (3)), assuming the pores have a cylindrical geometry.

(3) $$r_{pm}=\frac{2W}{100_{\rho_{a}}A}$$

where W is the absorption percentage, $\rho_{a}$ is the adsorbed phase density, and A is the specific surface area evaluated by the BET method.

The differences observed between the values of the sorption capacities for the studied membranes are the result of the differences in their structure and morphology. In such investigations, the structure–permeability relationship is a key parameter, especially in the case of systems that present polar groups. Thus, by analyzing the experimental results, we can observe that the sorption capacity decreases as follows: PSFQ > CAP > 40PSFQ/10CAP/50PVDF > 25PSFQ/25CAP/50PVDF > PVDF. This variation can be explained by taking into account the influence of the charge density of the chains due to the presence of quaternary ammonium groups as well as the nature of the solvent used. The effect of a solvent that forms structures with a large specific surface area and small pore sizes in the polysulfonic matrix is noteworthy. DMF and NMP are polar solvents which determine the mobility or relaxation of the polysulfonic chains, providing enough space for water molecules to integrate into the functionalized system. Also, the functional groups’ orientation during the electrospinning process is another parameter that influences the water vapor sorption capacities. Thus, after the electrospinning process, the orientation and distribution of the functional groups, depending on the solvent used, generate on the membranes’ surfaces interconnected pores of different sizes depending on the composition of the polymer mixture, the solvent, and the concentration (Table 3).

From the results presented in Table 3, it can be concluded that fibrous materials produced by the electrospinning method exhibit a larger surface area compared to PSFQ films [26]. Therefore, based on our findings, achieving a high-quality electrospun membrane for biomedical applications requires ensuring suitable physicochemical properties tailored to the specific requirements of the intended application. This can be achieved by chemical modifications, blending processes, and various techniques for processing solutions. Thus, through the research carried out, it was shown that it is the surface properties of the fibrous membranes obtained (PSFQ/CAP/PVDF) that dictate their functionality, performance, and, implicitly, their biological ability, not their specific surface.

### 3.3. Evaluation of the Applicative Potential of the Fibrous Membranes: Antimicrobial Activity

In recent years, scientific interest has shifted not only towards synthesizing new types of polymeric materials but also towards modifying existing polymers and their properties to meet the demands of emerging applications, particularly in the biomedical field [87]. Furthermore, advancements in biomedicine and other scientific disciplines have unveiled new challenging discoveries, prompting a need for objective evaluations of novel techniques. In this context, the application of polysulfone materials has been primarily focused on blood-contact devices, including hemodialysis, hemodiafiltration, and hemofiltration as membranes [88]. Additionally, polysulfone materials have found utility in cell- and tissue-contact devices, such as bioreactors based on hollow-fiber membranes [89,90], as well as in nerve regeneration applications facilitated by semipermeable hollow PSF membranes [91,92]. In an attempt to design a polymeric material to serve in the hemodialysis (HD) process, which in recent years has clearly proven that it cannot be considered a simple process, whereby blood and dialysate are separated by an inert semipermeable membrane, researchers have resorted to the creation and modeling of new membrane materials with increased efficiency in medical therapies that limit or reduce negative effects during HD therapy, especially the harmful effects of free radicals [93,94,95]. In this sense, bioactive fibrous membranes functionalized with antioxidants, such as α-tocopherol (α-TCP, 5 mg/mL concentration) and α-lipoic acid (ALA, 40 mg/mL), were prepared by directly incorporating them into the solvent used for polymer dissolution. At the same time, along with the cell–material interaction, antimicrobial activity plays a major role, with inhibitory effects representing an indicator in establishing the relationship between the surface structure properties of a polymer and membrane functionality [96,97]. In particular, the evaluation of antimicrobial activity using *Staphylococcus aureus* and *Escherichia coli* microorganisms indicates the potential for medical use and contributes to widening the studied polysulfonic systems’ applicability as membranes in hemodialysis processes.

The antimicrobial efficacy of the membranes obtained was determined using the contact time technique, and the results were influenced by the sample tested, the contact time, and the microbial culture tested (Table 4).

**Figure 10:**
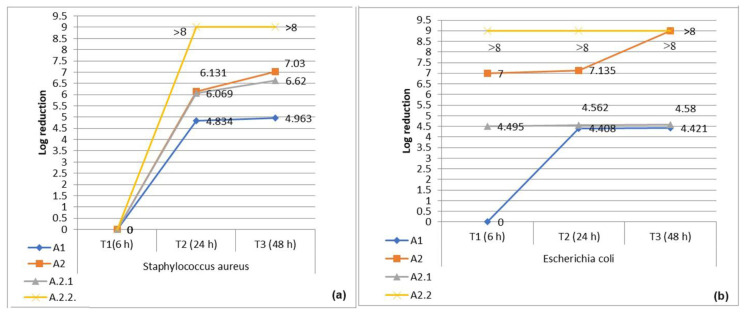
Logarithmic reduction in the number of bacterial cells (CFU) after contact times of 6 h (T1), 24 h (T2), and 48 h (T3) with samples A1, A2, A2.1, and A2.2: (**a**) *S. aureus* and (**b**) *E. coli*.

Depending on the contact time (6/24/48 h), the polysulfone materials showed antimicrobial activity against both *Staphylococcus aureus* (a Gram-positive species) and *Escherichia coli* (a Gram-negative species). The comparison of the antimicrobial efficacy of the two membrane structures based on polysulfones showed that sample A2 (40PSFQ/10CAP/50PVDF) exhibited better inhibition than sample A1 (25PSFQ/25CAP/50PVDF), but without significant differences, because after 24 h of contact, the percentage of microbial reduction was 99.999% (A1)–100% (A2) against *Staphylococcus aureus* and 99.996% (A1)–100% (A2) against *Escherichia coli* (Figure 10). This antimicrobial activity was maintained even after 48 h of contact, demonstrating the microbicidal effect of the matrix compounds (Figures S3–S5, Supplementary Material). The functionalization of the matrix in sample A2 with α-tocopherol (sample A2.1) and α-lipoic acid (sample A2.2) had no significant effect on its antimicrobial efficacy, as the logarithmic reduction was the same when tested against *Staphylococcus aureus* (100%/24–48 h) and ranged between 99.997 and 100% (24–48 h) in tests against *Escherichia coli* (Figure 10). These results demonstrate that the introduction of antioxidants has an important role in the hemodialysis process but does not necessarily have an effect on biological activity; instead, the polymer matrix-electrospun membranes based on polysulfones (PSFQ/CAP/PVDF) potentiate their functionality, sustainability, and performance, minimizing cell growth and proliferation.

In summary, the bactericidal efficacy of novel polysulfone materials with unique microarchitectures hinges on their capacity to strike an equilibrium between biological selectivity and membrane functionalization. Assessing the applicative potential of these membranes, particularly in terms of antimicrobial activity, underscores their exceptional performance and positions them as promising candidates for further exploration in the targeted domain. This affirmation not only highlights their current effectiveness but also encourages ongoing research efforts to unlock their full potential in addressing appropriate challenges in relevant fields.

## 4. Conclusions

This study focused on the development of novel membrane materials tailored for potential applications across various biomedical fields. Hence, an innovative approach that presents the potential to yield sustainable materials capable of addressing the growing demand in specific fields involves the development of PSFQ/CAP/PVDF blends, achieved through the control and enhancement of their properties. In this context, the ability to obtain the desired electrospun materials with well-established morphological and surface properties was highlighted by the relationship between the properties of the multicomponent systems’ solutions and their electrospinnability. According to research on solution rheology in relation to the electrospinning conditions, it was found that increasing the concentration of polymer solutions and their blending can enhance the solution’s conductivity/flexibility and that the stability of the solution jet during the process is quite good at a concentration of 40 g/dL. In this sense, the semidilute unentangled and semidilute entangled concentration domains were delimited. Although the scaling predictions between the concentration and the specific viscosity were different for the three types of polymer solutions, they are in accordance with data from the literature. Moreover, we found that the minimum proportion of polyelectrolyte (PSFQ) in the solution composition should not be less than 40 wt.%, and the optimum ratio required to obtain fibers with the best morphological and structural characteristics corresponds to a 50/50 (*v*/*v*) DMF/NMP composition in the mixture. Thus, as a result of these parameters, the SEM images demonstrate that the electrospinning of solutions with a concentration of 40 g/dL enables the achievement of uniform, continuous, bead-free fibers. On the other hand, the rheological response given by the viscoelastic moduli (G′ and G″) confirms the point where the stress-resistant and stable entanglement network stores enough elastic energy so that it leads to obtaining fibers instead of drops. As a result, it was observed that the surface features of the materials under study are significantly influenced by the specific properties of the polymers (PSFQ, CAP, and PVDF), as well as the choice of solvents utilized in the process. Moreover, the FTIR spectra of the fibrous membranes highlight different macromolecular rearrangements depending on the solvents/solvents’ mixture properties as a result of the functional groups’ orientation during the electrospinning process.

The practical applicability of the composite membranes (PSFQ/CAP/PVDF) in future biomedical applications is based on the good relationship between the structure and surface properties and their functionality. Thus, the bacterial adhesion on the electrospun membranes’ surfaces using *E. coli* and *S. aureus* microorganisms was tested. By combining the electrospinning technique with the antimicrobial properties of the polymers, a new strategy for the development of advanced materials that maximize the inactivation of bacterial cells was generated.

Through the careful control and enhancement of the properties of these membranes, we have highlighted their ability to meet the requirements for various biomedical applications. However, it is crucial to acknowledge the limitations and challenges associated with the current research findings. While our study has shown promising results at the laboratory scale, addressing scalability remains crucial. Further research is needed to optimize the electrospinning process for large-scale production, focusing on factors like process control and equipment scalability. Additionally, streamlining the optimization process to ensure reproducibility on a larger scale is imperative. Material compatibility and safety considerations are paramount for practical biomedical applications, necessitating further evaluation to assess the environmental impact. Despite these challenges, the findings of this study demonstrate the potential of composite membranes (PSFQ/CAP/PVDF) for various biomedical applications. The superior properties acquired, including enhanced flexibility, hydrophobicity, specific molecular microarchitecture, and controlled porosity, make these membranes highly desirable for biomedical use. Therefore, there is a strong incentive for the continued exploration and utilization of these membranes in the biomedical domain.

## Figures and Tables

**Table 1:** Electrospinning conditions and the average fiber diameters obtained from polymer solutions with a concentration of 40 g/dL.

Sample	Electrospinning Conditions	Average Fiber Diameters (µm)
PSFQ in DMF	22.1 kV ^a^, 10 µL/min ^b^, 20 cm ^c^, 29% ^d^, 25.3 °C ^e^	0.110 ± 0.045
CAP in DMF PVDF in NMP	21.8 kV ^a^, 20 µL/min ^b^, 20 cm ^c^, 30% ^d^, 24.3 °C ^e^ 23.8 kV ^a^, 50 µL/min ^b^, 20 cm ^c^, 39% ^d^, 25 °C ^e^	0.105 ± 0.046 0.073 ± 0.031
25PSFQ/25CAP/50PVDF in 50/50 DMF/NMP	24.1 kV ^a^, 10 µL/min ^b^, 16 cm ^c^, 41% ^d^, 22.4 °C ^e^	0.111 ± 0.049
40PSFQ/10CAP/50PVDF in 50/50 DMF/NMP	24.2 kV ^a^, 5 µL/min ^b^, 20 cm ^c^, 31% ^d^, 24 °C ^e^	0.121 ± 0.047

^a^ voltage; ^b^ the flow rate of the solution; ^c^ the distance between the tip of the needle and the collector; ^d^ relative humidity (RH); ^e^ temperature.

**Table 3:** Surface parameters evaluated based on the sorption/desorption isotherms of the studied materials: water vapor sorption capacity (W), average pore size (r_pm_), and BET data (specific area, A; monolayer).

Sample	W (%)	r_pm_(nm)	BET Data	
			A (m^2^/g)	Monolayer (g/g)
PSFQ/DMF	21.35	2.03	210.54	0.060
	3.69 *		64.15 *	0.018 *
CAP/DMF	17.68	2.02	174.66	0.049
PVDF/NMP	0.430	1.73	2.91	0.0008
25PSFQ/25CAP/50PVDF in 50/50 DMF/NMP	5.21	1.61	64.77	0.0184
40PSFQ/10CAP/50PVDF in 50/50 DMF/NMP	5.51	1.60	68.93	0.0196

* According to previous results obtained for PSFQ films [26].

**Table 4:** Antimicrobial activity of bioactive fibrous membranes against standardized microbial species.

Bacteria Species	Times	A1 (CFU/mL/ 1 cm^2^)	A2 (CFU/mL/ 1 cm^2^)	A2.1 (CFU/mL/ 1 cm^2^)	A2.2 (CFU/mL/ 1 cm^2^)
*S. aureus*	Control sample	1.5 × 10^8^	1.5 × 10^8^	1.5 × 10^8^	1.5 × 10^8^
	T1 (6 h)	UQ	UQ	UQ	UQ
	Log reduction	0	0	0	0
	% reduction	0	0	0	0
	T1 (24 h)	21.96 × 10^2^	11.10 × 10^1^	12.8 × 10^1^	0 × 10^0^
	Log reduction	4.83	6.13	6.07	I
	% reduction	99.999	100	100	100
	T1 (48 h)	16.32 × 10^2^	1.4 × 10^1^	3.6 × 10^1^	0 × 10^0^
	Log reduction	4.96	7.03	6.62	I
	% reduction	99.999	100	100	100
*E. coli*	Control sample	1.5 × 10^8^	1.5 × 10^8^	1.5 × 10^8^	1.5 × 10^8^
	T1 (6 h)	UQ	1.5 × 10^1^	48 × 10^2^	32.18 × 10^2^
	Log reduction	0	7	4.49	I
	% reduction	0	100	99.997	100
	T1 (24 h)	58.66 × 10^2^	1.1 × 10^1^	41.13 × 10^2^	0 × 10^0^
	Log reduction	4.41	7.13	4.56	I
	% reduction	99.996	100	99.997	100
	T1 (48 h)	56.84 × 10^2^	0 × 10^0^	30.49 × 10^2^	0 × 10^0^
	Log reduction	4.42	I	4.58	I
	% reduction	99.996	100	99.997	100

UQ—unquantifiable; 0—there is no bacterial growth; I—infinity (microbicide).

## Data Availability

Data are contained within the article and Supplementary Materials.

## Supplementary Materials

The following supporting information can be downloaded at https://www.mdpi.com/article/10.3390/polym16111503/s1, Figure S1. Rheological curves: dynamic viscosity (η) as a function of shear rate (γ̇) at 25 °C for PSFQ in NMP at concentrations between 15 and 35 g/dL; Figure S2. Dependence of specific viscosity on concentration for CAP in DMF and PVDF in NMP; Figure S3. Control samples; Figure S4. Testing the antimicrobial activity of membranes based on polysulfones:A1 (25PSFQ/25CAP/50PVDF), A.2 (40PSFQ/10CAP/50PVDF), A2.1 (40PSFQ/10CAP/50PVDF + α-TCP), and A2.2 (40PSFQ/10CAP/50PVDF + ALA) after 6/24/48 h of contact with a *Staphylococcus aureus* suspension (1.5 × 10^8^ CFU); Figure S5. Testing the antimicrobial activity of membranes based on polysulfones: A1 (25PSFQ/25CAP/50PVDF), A.2 (40PSFQ/10CAP/50PVDF), A2.1 (40PSFQ/10CAP/50PVDF + α-TCP), and A2.2 (40PSFQ/10CAP/50PVDF + ALA) after 6/24/48 h of contact with a *Escherichia coli* suspension (1.5 × 10^8^ CFU).
