# Supplementary material for: Electrospun Membranes Based on Quaternized Polysulfones: Rheological Properties–Electrospinning Mechanisms Relationship
Source: Polymers (Basel). 2024 May 25;16(11):1503. doi: 10.3390/polym16111503 (PMC11174964; doi:10.3390/polym16111503)
Supplement: Supplementary file 1 [file polymers-16-01503-s001.zip › polymers-2986688-supplementary.pdf]

Supplementary Materials

Electrospun Membranes Based on Quaternized Polysulfones: Rheological Properties–Electrospinning Mechanisms Relationship

Anca Filimon [1,*], Diana Serbezeanu [1], Adina Maria Dobos [1], Mihaela Dorina Onofrei [1], Alexandra Bargan [1], Daniela Rusu [1] and Cristina Mihaela Rimbu [2]

[1] "Petru Poni" Institute of Macromolecular Chemistry, Grigore Ghica Alley 41A, 700487 Iasi, Romania; diana.serbezeanu@icmpp.ro (D.S.); necula.adina@icmpp.ro (A.M.D.); mihaela.onofrei@icmpp.ro (M.D.O.); anistor@icmpp.ro (A.B.); rusu.daniela@icmpp.ro (D.R.)
[2] Department of Public Health, University of Life Science Iasi, 8 Mihail Sadoveanu Alley, 707027 Iasi, Romania; crimbu@yahoo.com
* Correspondence: afilimon@icmpp.ro

Associated content includes rheological data and the performed tests to quantitatively determine the antimicrobial efficacy of the evaluated materials against the control sample using the contact time technique.

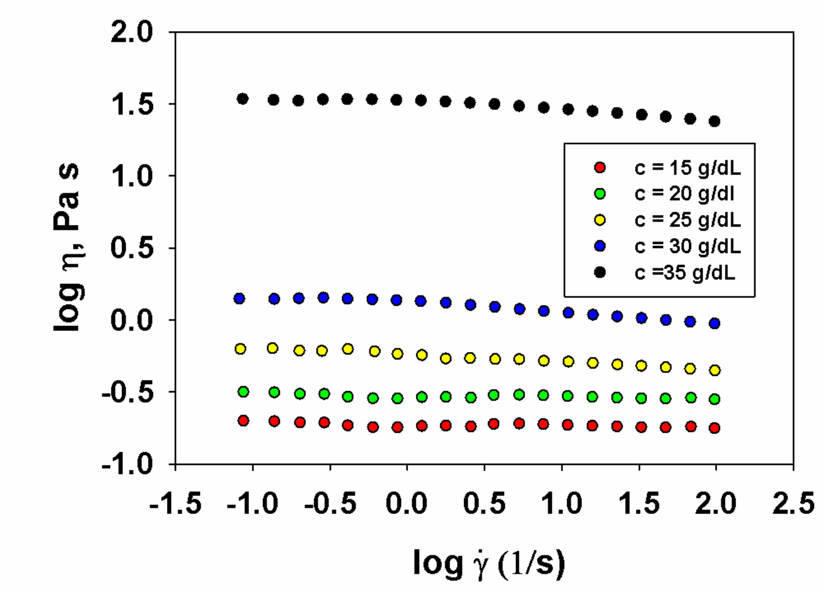

Figure S1. Rheological curves: dynamic viscosity (η) function on shear rate ($\dot{\gamma}$) at 25 °C for PSFQ in NMP at concentrations between 15-35 g/dL.

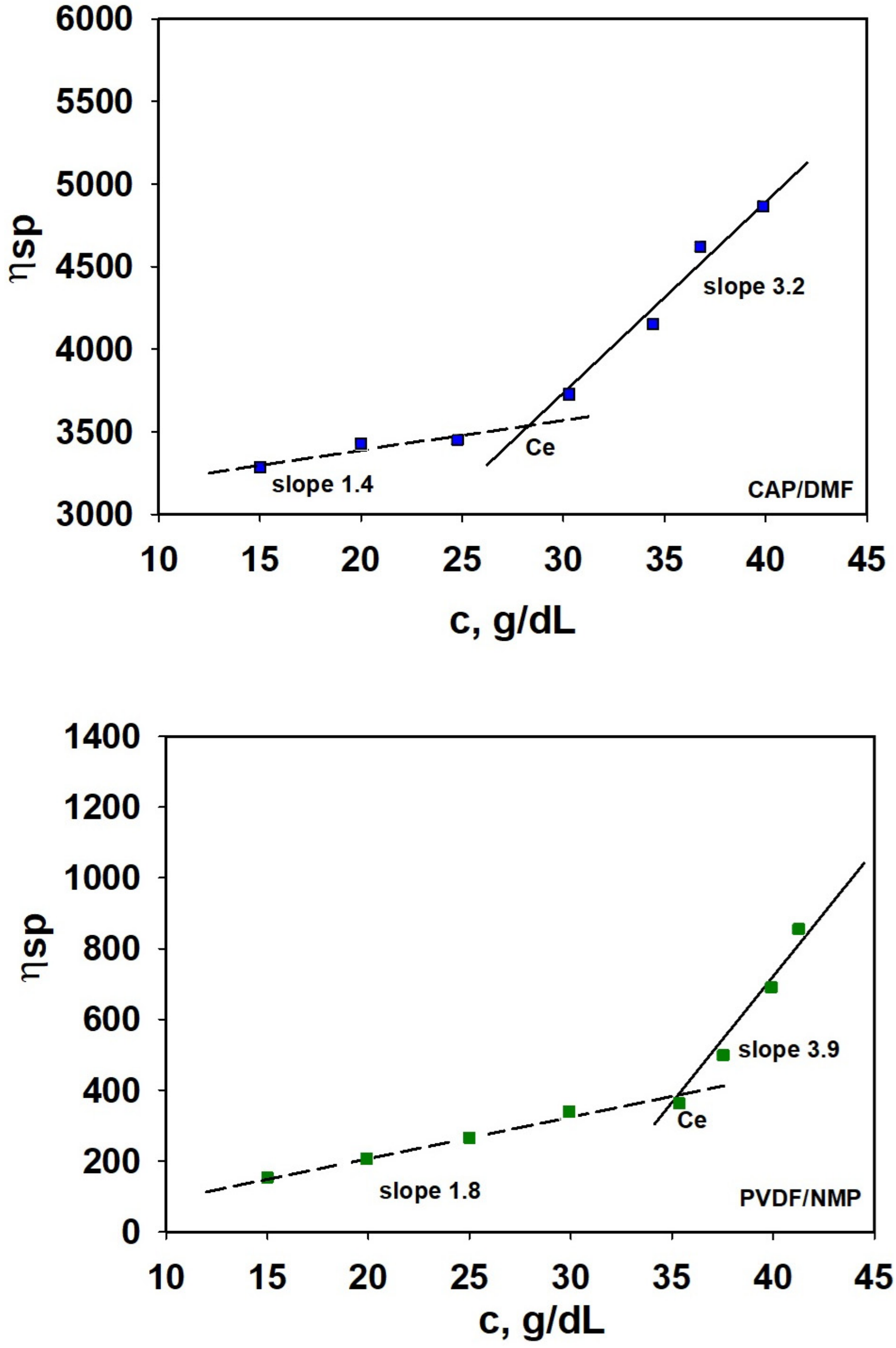

Figure S2. Dependence of specific viscosity on concentration for CAP in DMF and PVDF in NMP.

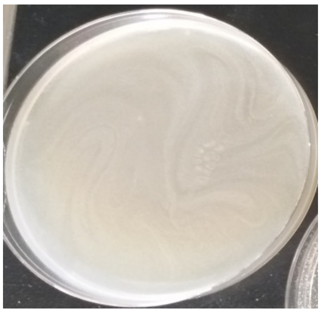	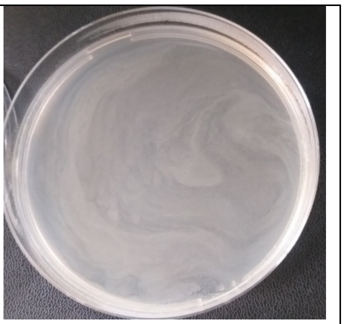
***Staphylococcus aureus* -** **(1.5 x 10^8CFU/mL)**	***Escherichia coli* –** **(1.5 x 10^8 CFU/mL)**

Figure S3. Control samples.

	A1	A2	A2.1	A.2.2
6 h	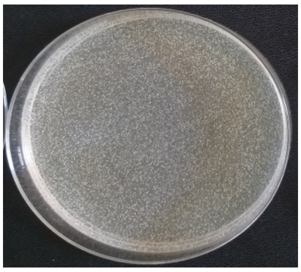	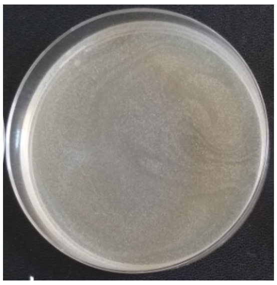	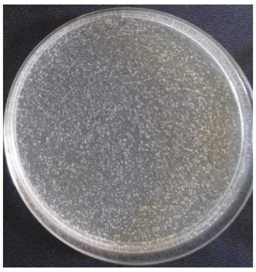	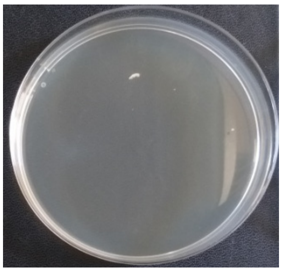
24 h	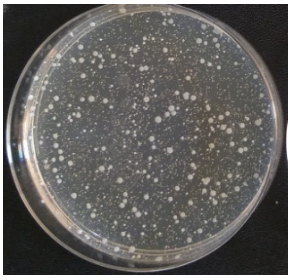	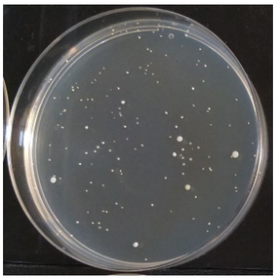	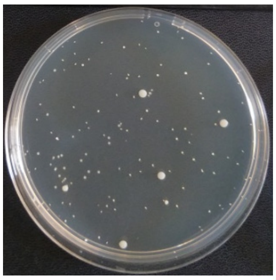	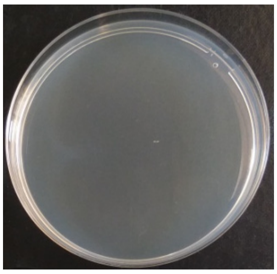
48 h	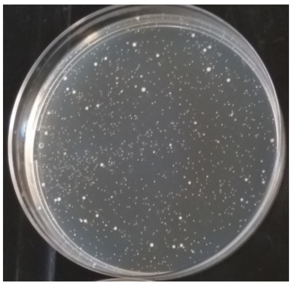	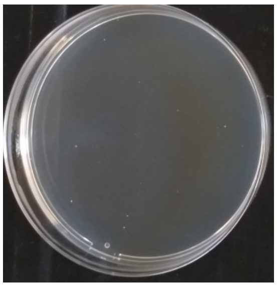	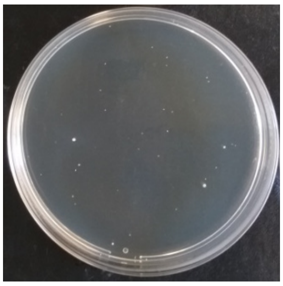	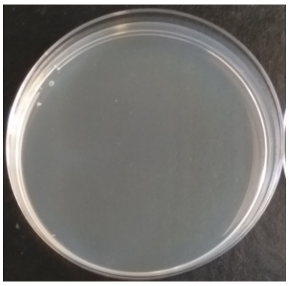
	Staphylococcus aureus ATCC 25923			

Figure S4. Testing the antimicrobial activity of membranes based on polysulfones: A1 (25PSFQ/25CAP/50PVDF), A.2 (40PSFQ/10CAP/50PVDF), A 2.1. (40PSFQ/10CAP/50PVDF +

α-TCP), A2.2 (40PSFQ/10CAP/50PVDF + ALA) after 6/24/48 hours of contact with a *Staphylococcus aureus* suspension (1.5 x 10^8 CFU).

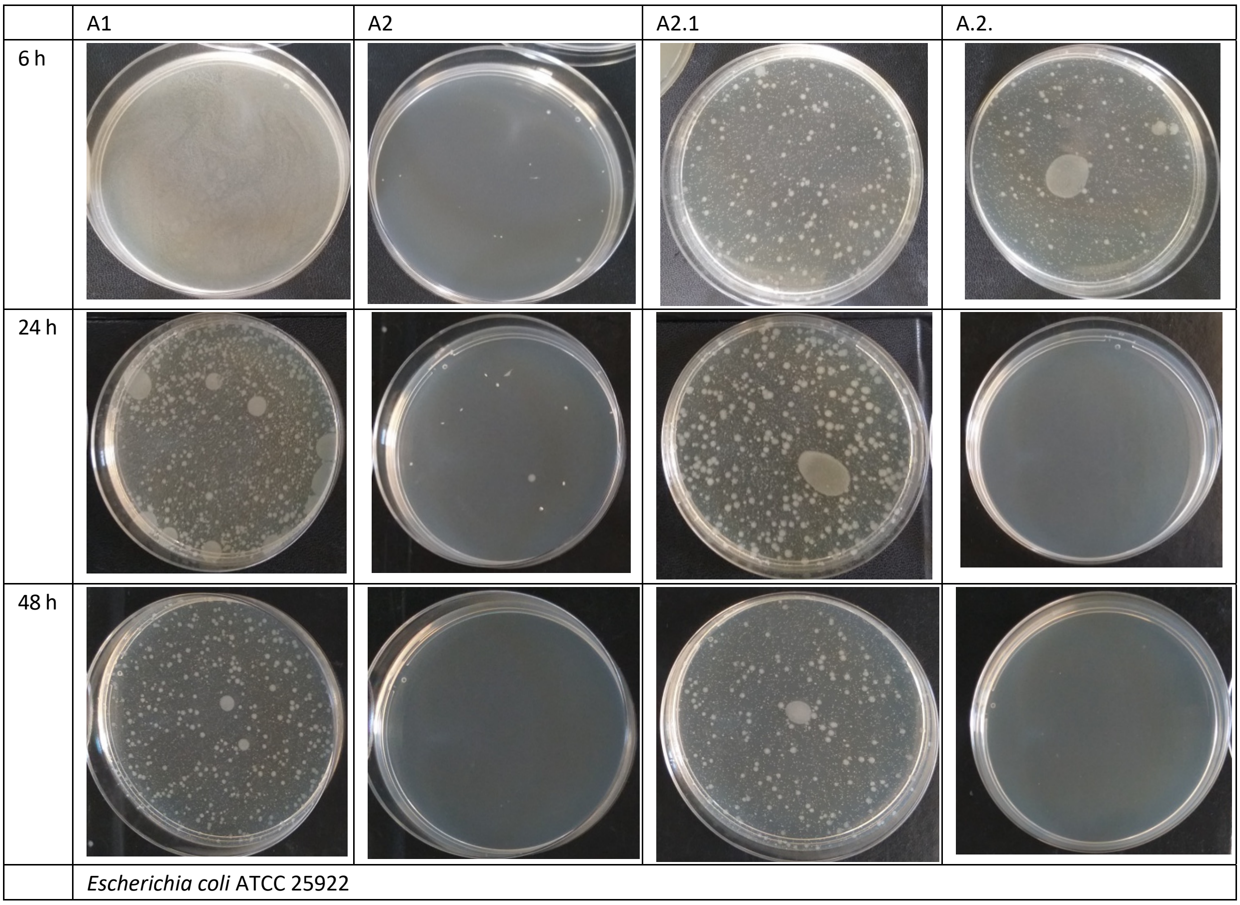

Figure S5. Testing the antimicrobial activity of membranes based on polysulfones A1 (25PSFQ/25CAP/50PVDF), A.2 (40PSFQ/10CAP/50PVDF), A2.1 (40PSFQ/10CAP/50PVDF + α-TCP), A2.2 (40PSFQ/10CAP/50PVDF + ALA) after 6/24/48 hours of contact with a *Escherichia coli* suspension (1.5 x 10^8 CFU).
